# The Relationship between Neighborhood Environment and Child Mental Health in Japanese Elementary School Students

**DOI:** 10.3390/ijerph17155491

**Published:** 2020-07-29

**Authors:** Rikuya Hosokawa, Toshiki Katsura

**Affiliations:** 1 Graduate School of Medicine, Kyoto University, Kyoto 606-8507, Japan; 2 Faculty of Health Care, Tenri Health Care University, Tenri 632-0018, Japan; tkatsura@tenriyorozu-u.ac.jp

**Keywords:** neighborhood environment, child mental health, behavioral problems, prosocial behavior, elementary school students, middle childhood, socioeconomic status

## Abstract

Limited research has examined the relationship between neighborhood environment and mental health outcomes in elementary school students (middle childhood). In countries with high relative poverty, little is known about how neighborhood conditions are related to children’s health after controlling for family socioeconomic status; thus, it is necessary to distinguish the particular neighborhood characteristics relevant to behavioral risk in children, independent of socioeconomic position. Using a self-report survey completed by parents, we assessed neighborhood environment characteristics, children’s behavioral outcomes, and family socioeconomic status in fourth grade students from Nagoya, in Aichi prefecture, Japan (*n* = 695). A multiple linear regression was conducted to evaluate to what extent neighborhood characteristics predict child behaviors, after adjusting for socioeconomic variables. Greater aesthetic quality, walkability, accessibility of healthy foods, safety, and social cohesion were inversely linked to children’s behavioral problems and positively linked to social competence, suggesting that quality of living environment may affect behavioral outcomes in children, even after controlling for socioeconomic factors. Developing a quality environment that matches these characteristics may minimize the negative impact of a family’s socioeconomic distress and is likely to aid socioeconomically disadvantaged parents and their children. Thus, policies and programs that enhance the neighborhood environment for socioeconomically disadvantaged families should be promoted.

## 1. Introduction

There is considerable evidence that neighborhood conditions affect a range of child developmental outcomes [1,2,3], including the well-being and behavior of developing children. Additionally, there has been an increase in attention regarding the role of neighborhoods and residential environments in explaining differences in health outcomes [4]. While most studies have focused on relationships between the neighborhood environment and children’s behavioral outcomes in early childhood [5,6,7], less research has been devoted to these relationships in elementary school-aged children (middle childhood).

Age is important in the context of child development, as there are various theoretical childhood stages that can cause children to be sensitive to their surroundings [8]. For example, early childhood represents a very sensitive period of development in terms of the various child-rearing environmental factors surrounding children [9]. However, some evidence implies that older children may be more vulnerable to environments outside the home [10]. From the life-course perspective, ecological embeddedness intensifies with age, as children grow increasingly independent from their families [8]. While the family home is arguably the most influential proximate environment on a young child’s development, as well as a place they occupy for a prolonged time, older children spend more time outside the home (i.e., in their neighborhood), compared to younger children [1]. Accordingly, older children could be more susceptible to neighborhood influences.

Previous research has analyzed the relationship between neighborhood environment and behavioral outcomes in adolescents [1,11]. Findings indicate that residential surroundings may become more pertinent during adolescence due to peer pressure influence and a growing engagement with neighborhoods, schools and other similar institutions [10]. In addition, for older children, neighborhood effects are likely to function more directly through contact with institutions that provide services and information [1].

Middle childhood is an important developmental transition from early childhood to adolescence, as children during this stage begin to develop their ecological network ties [12]. For instance, children’s cognitive and non-cognitive competences develop rapidly as they transition from childhood to adolescence [8]. Notably, during middle childhood, children mature both physically and emotionally, and increase their interactions with diverse sets of individuals, demands, and notions [13]. The level of independence from their family home environment may combine with greater social connections in their neighborhoods to increase children’s sensitivity to circumstantial conditions. Arguably influenced by the neighborhood environment, the development of children during middle childhood has long-term effects on social adjustment and functioning in later adolescence and adulthood [12]. Nevertheless, limited research has been conducted to examine the effect of neighborhood conditions on children in middle childhood as compared with young children and adolescents. Therefore, examining the relationship between neighborhood condition and developmental outcomes in middle childhood is warranted.

Furthermore, although the relationship between the neighborhood environment and children’s behavioral outcomes has been extensively covered in Western countries [1,2,3], there have been limited attempts to examine this association in Asian countries, including Japan. Notably, there are prospective differences in geographical and cultural backgrounds between Asian and Western countries [14]. For instance, over the past few decades, several countries in East Asia have faced rapid industrialization, resulting in notable changes to living environments and living standards [15,16]. This is especially true for Japan, which led this East Asian industrialization progression. Although Japan has a fairly extensive history of economic development and high living standards, there are currently sizable differences in health status between children from rich and poor families. For example, the Organization for Economic Co-operation and Development (OECD) has shown that the risk of relative poverty for families with children has risen in many OECD countries [17]. Relative child poverty rates have increased in almost two-thirds of OECD countries since the great recession of 2007–2008. In particular, Japan’s rate of child relative poverty is above the OECD average. The risk of relative poverty in Japan has increased despite its high gross domestic product, with a child/poverty ratio of about 14% in the mid-2000s, exceeding the OECD average of 12%. One in seven children live on less than half of what is considered the average living standard.

At one point, Japan was regarded as a country that sustained an egalitarian society and exhibited strong social cohesion and solidarity [18,19]. However, since the collapse of the 1990s economic bubble, an increase in socioeconomic inequalities has overwhelmed the current population, altering social relations; thus, Japanese society has entered a new reality of income and education inequality, and job insecurity.

Importantly, inequality in family socioeconomic status (SES) can affect child development [20,21]. The home environment, which includes family SES, is the proximate environmental influence on child development and is the place where children spend a prolonged period of time. Research indicates that lower SES can affect children’s developmental outcomes; for instance, the quality of the home environment may account for a substantial portion of the effects of family income on children’s developmental outcomes [22,23,24]. Indeed, families with higher SES have greater access to financial, social, and human capital than lower SES families, and these resources contribute to the successful development of children. However, for those with a high risk of relative poverty, little is known about the ways in which the living environment is related to children’s health and which living environments play a more crucial role in that relationship. From the perspective of differences in geographical and cultural backgrounds, it is important to identify the neighborhood environment and its association with behavioral development among Japanese children so that we can gain a deeper understanding of the relationships among these variables.

Various characteristics of the neighborhood environment have been evaluated from several perspectives. For instance, neighborhood social cohesion and safety have been well documented as factors that may determine children’s health outcomes [25,26,27,28]. Indeed, neighborhood social cohesion and safety are important environmental resources, likely to protect against neighborhood-level risk factors. Neighborhood social cohesion is characterized by mutual trust and social support. Safety is characterized by social and traffic safety such as crime and high traffic exposure. While neighborhoods provide significant contexts for understanding children’s development and behavior, difficulties have arisen regarding attempts to develop measures to examine ecological settings at the neighborhood level. Indeed, the importance of studying the main effect of the environment, and interactions between environmental and individual factors, has been repeatedly emphasized in epidemiology. However, the assessment of ecological settings remains in its early stages. Nevertheless, using survey data, Mujahid et al. revealed the feasibility of measuring constructs that differ across geographic areas [29]. In their scale, which was found to be valid and reliable, Mujahid and colleagues propose that seven social and physical neighborhood environment features may be associated with health risk, namely: violence, safety, aesthetic quality, walking environment, availability of healthy foods, social cohesion, and activities with neighbors. Even though environmental characteristics appear to significantly influence the development of children’s behavior, few studies have explored the link between multifaceted environmental features and children’s behavior in middle childhood. Thus, exploring whether neighborhood features impact child behavioral outcomes is necessary to identify neighborhood factors that can potentially be modified and those that are influential, as doing so may contribute to more effective community-level interventions and change.

In summary, while environmental characteristics appear to play a role in determining behavioral outcomes in children, limited research has analyzed the association between neighborhood conditions and behavioral outcomes in middle childhood or in Asian countries such as Japan. Moreover, in countries with relatively high poverty, little is known about how neighborhood conditions are related to children’s health after controlling for the effect of individual family SES; thus, specific features of neighborhoods relevant to behavioral risk in children need to be identified, independent of SES. Additionally, few studies have explored the connection between multifaceted environment features (i.e., violence, safety, aesthetic quality, walking environment, availability of healthy foods, social cohesion, and activities with neighbors) and children’s behaviors in middle childhood. Given that specific features of neighborhoods are understudied, understanding the role of neighborhood characteristics and its association with behavioral outcomes is important for designing policies and to lessen or eliminate socioeconomic disparities in health. Therefore, the present study examines the association between the characteristics of the surrounding environment and the behavioral outcomes of Japanese children in middle childhood, after controlling for the effect of individual family SES.

## 2. Materials and Methods

### 2.1. Procedure and Participants

The present study used data from a prospective cohort study that examined child development, adjustment, and their influencing factors. The study was conducted in Nagoya, in Aichi prefecture, Japan. In 2014, participants were recruited from 130 kindergarten and nursery schools in Nagoya city. To recruit participants, self-reported questionnaires were distributed to all parents of five- and six-year-old preschool children in their final grade of kindergarten or nursery school. Parents gave written informed consent for their children to participate. An annual survey was conducted as the children grew older, and their development was followed from preschool to junior high school. A baseline measure was established via the first wave that occurred in 2014, during which we obtained the addresses of the participants.

The present study uses data from the fifth wave of data collection, which was conducted in 2018. For this wave, one questionnaire per child was distributed to the children’s parents. The parents then completed the questionnaire and returned it to the researcher by mail. The self-report questionnaire was distributed to participants we considered suitable for the follow-up survey; in this case, they were parents of nine- and ten-year-old children (*n* = 1490) in the fourth grade of elementary school. Of the 1490 questionnaires distributed, 778 completed questionnaires were returned (response rate: 52.2%). To accurately determine the relationship between neighborhood environment and child adjustment and behavioral outcomes, we excluded the following subjects from analysis: (1) children diagnosed with behavioral problems and (2) children whose parents did not return the completed questionnaire. Of the 778 children whose parents completed the questionnaire, 695 (89.3%) of them met our inclusion criteria. We compared returning participants with non-returning participants on SES demographic features at baseline, and chi-squared tests were performed. As it is difficult to compare household income over time, parental education level was used as a proxy for SES. Results showed a significant difference between the groups’ maternal education level, with 36.8% of returning participants reporting below “More than four years at college/university”, while only 25.8% of non-returning participants reported this level (*p* < 0.001). There was also a significant difference in terms of paternal education level, with 54.8% of returning participants reporting below “More than four years at college/university,” while only 44.7% of non-returning participants reported this level (*p* < 0.001). In other words, those with a higher SES tended to have a higher return rate, while those with a lower SES tended to have a lower return rate.

### 2.2. Measures

#### 2.2.1. Outcome Variable: Child Behavior

The Strengths and Difficulties Questionnaire (SDQ) is a behavioral screening tool designed to assess child well-being [30,31]. In the present study, parents completed the SDQ on behalf of their children. The SDQ is a 25-item scale that uses a three-point Likert scale, with responses ranging from not true (0) to certainly true (2). The SDQ measures social competence and both externalizing and internalizing behaviors. It comprises five subscales: emotional symptoms (five items); conduct problems (five items); hyperactivity (five items); peer problems (five items) and prosocial behavior (five items; see Table S1). In the SDQ, a difficult behavior score is calculated by summing all the scores, with higher scores representing increased behavioral difficulties and an increased probability of the child having a mental disorder. Based on the reference of Goodman et al., we combined the conduct problems and hyperactivity-inattention subscales into an externalizing problems scale; additionally, the peer problems and emotional problems subscales were merged into an internalizing problems scale [32], all of which were later analyzed. These scales show satisfactory internal consistency [30,31,32] and the SDQ has been cross-culturally validated for the Japanese context [33]. To assess community-based data and properties for the Japanese version of the questionnaire, Matsuishi et al., assembled and examined parent ratings of a total of 2899 Japanese children with ages ranging from 4–12 years. The parent SDQ has been validated for a Japanese-speaking population and recommended classification of normal, borderline, and clinical ranges have been outlined for each of the five scales, along with the original English questionnaire. The Japanese version was found to be approximately as dependable and useful as the original English questionnaire. The SDQ’s reliability and validity among Japanese school children has been previously recorded [34,35]. In this study, Cronbach’s alpha coefficients were 0.76 for the externalizing problems subscale, 0.70 for the internalizing problems subscale, and 0.72 for the prosocial behaviors subscale (Table 1).

#### 2.2.2. Explanatory Variable: Neighborhood Characteristics

The Neighborhood Scale was used to assess neighborhood resources [29]. For the purposes of this scale, a neighborhood is defined as the area approximately one mile around the child’s home. This scale assesses seven dimensions of the neighborhood environment, including aesthetic quality (five items), walking environment (seven items), availability of healthy foods (three items), perception of safety (three items), violence (four items), social cohesion (four items), and activities with neighbors (five items; see Table S2). Aesthetic quality refers to the pleasantness of surroundings, which may include interesting and attractive well-maintained buildings and homes, and gardens without noise, graffiti, and litter. Walking environment does not just refer to walkability; a neighborhood provides many other opportunities to exercise. Neighborhood facilities such as sports clubs offer a number of opportunities to be physically active. Availability of healthy foods refers to the accessibility to a large selection of fresh and quality fruits and vegetables. Safety refers to the perception of being safe, a sense of the absence of violence, and of being safe from crimes. Violence refers to fights, sexual assault/rape, and robbery/mugging in the neighborhood environment. Social cohesion describes a mutual trust and social support among community members, including willingness to help neighbors, getting along with each other, and sharing the same values. Activities with neighbors refers to favoring our neighbors, asking neighbors for advice on personal things, and visiting the homes of neighbors or speaking to them. For the domains of safety, aesthetic quality, walking environment, availability of healthy foods and social cohesion, responses were provided on a five-point Likert scale, ranging from strongly disagree (1) to strongly agree (5), with higher scores reflecting higher perceptions of aesthetic quality, walking environment, availability of healthy foods, safety and social cohesion. For violence and activities with neighbors, responses were provided on a four-point Likert scale, ranging from never (1) to often (4). For these domains, responses were reverse scored to increase clarity of interpretation so that higher scores implied greater neighborhood violence and more activities with neighbors. All scores were calculated by averaging all items in the scale. In previous research, the scales exhibited adequate internal consistency [29]. In the present study, Cronbach’s alpha coefficients ranged from 0.69 to 0.90 (Table 1).

#### 2.2.3. Confounding Variables

We collected self-reported information regarding the child’s gender, annual household income, and the mother’s and father’s education level. The primary confounding variable associated with child behavior and neighborhood environment is the family socioeconomic circumstance, which includes annual household income and parental education level. Indeed, children from disadvantaged families tend to live in lower quality neighborhoods [36,37] and have greater behavioral problems [20,21]. In addition, a child’s gender plays an important role in behavioral outcomes; for instance, among the children who were exposed to adversity, prior studies show that males exhibited more externalizing problems than females, while females showed more internalizing problems than males [38,39]. In the present study, the socioeconomic variables were significantly associated with several neighborhood characteristics and child behavior variables (see Tables S3 and S4). In addition, a child’s gender was significantly associated with several child behavior variables (see Table S4). Consequently, in our adjusted model, we included indicators of family socioeconomic circumstances and the child’s gender as covariates to account for these confounds.

### 2.3. Ethics Statement

All children’s parents were informed of the process and objectives of this study and advised that they were under no obligation to partake in the baseline survey. Prior to participating in the study, the parents provided written informed consent on behalf of their children. In addition, in every survey, as with the baseline survey, researchers informed the children’s parents about the study’s objectives and procedures and advised that they were not obligated to participate through the description enclosed with the questionnaires. The study was conducted in accordance with the Declaration of Helsinki, and the protocol was approved by the Kyoto University Ethics Committee (E2322).

### 2.4. Statistical Analyses

IBM SPSS Statistics Version 26 was used for data analysis. Associations between neighborhood characteristics and child outcomes were evaluated using multiple linear regression. In particular, we measured to what extent neighborhood characteristics (i.e., aesthetic quality, walkability, availability of healthy foods, safety, violence, social cohesion, and activities with neighbors) predicted child behavior outcomes (i.e., externalizing and internalizing problems and prosocial behaviors). Regression analyses were structured as follows. In Model 1, the aforementioned neighborhood variables were entered into an unadjusted model for each outcome. In Model 2, the neighborhood variables were entered into an adjusted model that controlled for family SES (i.e., annual household income and parental education level) and child gender. Although several variables (neighborhood variables as explanatory variables and socioeconomic variables as control variables) were significantly associated with each other (see Table S5), multicollinearity was evaluated using the variance inflation factor, which was <2, indicating no problems with multicollinearity.

## 3. Results

### 3.1. Participant Characteristics

In total, 695 child subjects were analyzed. The average age of the participants was 10.11 years (SD = 0.47). Regarding gender, 49.5% of the subjects were males (*n* = 344) and 50.5% were females (*n* = 351). Socioeconomic characteristics are presented in Table 2. In terms of annual household income (1 USD ≈ 100 JPY), over half (50.9%) of the families earned less than 6,000,000 JPY (≈60,000 USD). The median age of the mothers was 41.89 (SD = 4.40), and nearly half (36.8%) of them had up to four years of college/university education (e.g., junior colleges and vocational schools). The median age of the fathers was 43.95 (SD = 5.62), and over half (54.8%) of them had at least four years of college/university or higher degrees.

Given that children from more disadvantaged families live in lower quality neighborhoods (resulting in a greater risk of behavioral problems), before conducting our main analysis, we analyzed associations among socioeconomic variables, neighborhood characteristics, and child behavioral outcomes. Results indicated that socioeconomic variables were significantly associated with neighborhood characteristics (see Tables S3 and S4). That is, children from more disadvantaged families (i.e., low annual household income and parental education level) tended to live in lower quality neighborhoods. In addition, the socioeconomic variables were significantly associated with several behavioral characteristics. More specifically, children from more disadvantaged families tended to exhibit a greater risk of behavioral problems (i.e., externalizing problem behaviors). Thus, we included these socioeconomic variables as covariates in additional analyses.

### 3.2. The Link between Neighborhood Characteristics and Externalizing Problems

The results of our multivariate analysis examining the relationship between neighborhood characteristics and externalizing problem behaviors are shown in Table 3. In Model 1, which was unadjusted, aesthetic quality (standardized coefficient (β) = −0.147, *p* < 0.001), walking environment (β = −0.104, *p* = 0.007), and social cohesion (β = −0.105, *p* = 0.006) were significantly and inversely associated with externalizing problems. In Model 2, which was adjusted for socioeconomic factors, aesthetic quality (β = −0.140, *p* < 0.001), walking environment (β = −0.108, *p* = 0.006), and social cohesion (β = −0.128, *p* = 0.001) were significantly and inversely associated with externalizing problems.

#### 3.2.1. The Link between Neighborhood Characteristics and Internalizing Problems

Regarding the relationship between neighborhood characteristics and internalizing problems, Table 4 demonstrates the results of our multivariate analysis. In Model 1, which was unadjusted, aesthetic quality (β = −0.179, *p* < 0.001), walking environment (β = −0.133, *p* = 0.001), availability of healthy foods (β = −0.116, *p* = 0.002), safety (β = −0.097, *p* = 0.011), and social cohesion (β = −0.108, *p* = 0.005) were significantly and inversely associated with internalizing problems. In Model 2, which was adjusted for socioeconomic factors, aesthetic quality (β = −0.162, *p* < 0.001), walking environment (β = −0.124, *p* = 0.002), availability of healthy foods (β = −0.089, *p* = 0.026), and social cohesion (β = −0.101, *p* = 0.010) were significantly and inversely associated with internalizing problems.

#### 3.2.2. The Link between Neighborhood Characteristics and Child Prosocial Behaviors

The results of our multivariate analysis examining the relationship between neighborhood characteristics and prosocial behaviors are shown in Table 5. In Model 1, which was unadjusted, aesthetic quality (β = 0.111, *p* = 0.004) and social cohesion (β = 0.085, *p* = 0.027) were significantly and positively associated with prosocial behaviors. In Model 2, which was adjusted for socioeconomic factors, aesthetic quality (β = 0.124, *p* = 0.003), walking environment (β = 0.096, *p* = 0.018), safety (β = 0.091, *p* = 0.022), and social cohesion (β = 0.104, *p* = 0.008) were significantly and positively associated with prosocial behaviors.

## 4. Discussion

This study aimed to assess the associations among neighborhood characteristics and the behavioral outcomes of Japanese children in middle childhood after controlling for the effect of family SES. Prior to conducting our main analysis, we analyzed associations among socioeconomic variables, neighborhood characteristics, and child behavioral outcomes. The socioeconomic variables were significantly associated with several neighborhood characteristics and child behavioral problems, similar to previous studies [20,21,36,37]. In summary, our results indicated that children from disadvantaged families tend to live in lower quality neighborhoods and have a higher risk of developing behavioral problems. Thus, we included socioeconomic variables as covariates in our adjusted models.

Our main analysis indicated that neighborhood characteristics were strongly associated with child behavioral outcomes after adjusting for family socioeconomic variables. Specifically, neighborhood aesthetic quality, walking environment, and social cohesion were significantly and inversely associated with externalizing behavioral problems. In addition, greater aesthetic quality, walking environment, availability of healthy foods, and social cohesion were significantly and inversely associated with internalizing behavioral problems. However, greater aesthetic quality, walking environment, safety, and social cohesion were significantly and positively associated with prosocial behaviors. Even after adjusting for the impact of family disadvantage at an individual level, specific neighborhood features were likely to impact behavioral outcomes in middle childhood.

These findings suggest that the features of one’s living neighborhood environment are associated with child mental health, independent of individual family socioeconomic factors. This is consistent with the previous studies focusing on early childhood, adolescence, and children from other countries (e.g., Western countries) [1,2,3].

Additionally, in the present study, greater aesthetic quality, walking environment, availability of healthy foods, safety, and social cohesion were negatively associated with children’s behavior problems and positively associated with prosocial behaviors. There are likely several possible mechanisms behind these associations. First, outdoor play and physical activity is one possible mechanism. Outdoor play and physical activity are related to social interaction, exploration, and stimulation in children. Importantly, the relationship between the neighborhood environment and child behavior may be mediated by child development facilitating behaviors, such as play and physical activity [40,41]. Play is crucial to child development as it contributes to the cognitive, physical, social, and emotional well-being of children [42,43].

Opportunities for outdoor play and physical activity affect children’s social and emotional competence through interaction and stimulation, self-guided exploration, and imaginative play [42,44,45]. Parks and playgrounds are ideal settings for children to play freely and actively outside of the home. Although defined with regards to the aspects of social composition, neighborhood structure carries strong ties to the physical features of neighborhoods. Building characteristics are included in the physical aspects potentially relevant to children’s behavioral outcomes. Several studies suggest that environmental features of buildings, such as neighborhood aesthetics, walkability, and accessibility to local destinations (including easy access to supermarkets, shops, and public facilities) are positively related to child’s play and physical activity [46,47,48,49]. The features of aesthetic quality, walking environment, and availability of healthy foods are likely to facilitate access to physical environments that are stimulating and pleasant (e.g., parks, playgrounds, recreational facilities). Such access has been linked to increased play and physical activity [50,51].

However, many studies have identified safety as the primary concern; a decrease in safety leads to a decrease in children’s outdoor activities. Features of unsafe neighborhoods, including exposure to violence and crime, are negatively associated with children’s outdoor activities [52,53]. Parents in disorderly neighborhoods are reluctant to allow their children to spend time outdoors. Parental concerns and anxiety regarding neighborhood safety has grown, resulting in parental restrictions that affect the opportunities of children to play outside while interacting with others locally [54,55,56,57,58]. When parents consider urban environments dangerous, they tend to severely restrict their child’s access to outdoor spaces as a result, confining their child to indoor environments instead. Conversely, parental reassurance concerning neighborhood safety, although grounded in real concern, can serve to expand the location and range of neighborhood activities. Indeed, children in middle childhood are starting to expand their ecological network ties outside the home [12]. Thus, for these children, it may be important to live in an area where they and their parents feel safe.

Another possible mechanism behind the association between environmental characteristics and behavioral features is attractiveness. In the present study, aesthetics had the strongest positive association between neighborhood environment and child behavioral outcomes. The aesthetics of an environment points to the pleasantness of the surroundings, including interesting and attractive well-maintained buildings and homes, as well as garden spaces free of noise, graffiti, and garbage [29,59]. Children are impacted by their environment and are sensitive to its aesthetic and sensual aspects. This includes a notable inclination to scenic places and routes with plants, shade, trees, and well-maintained green spaces [60,61]. Conversely, children have consistently rejected places that are neglected, overgrown, or abandoned [62]. In studies targeting adults, the attractiveness of the environment, including an absence of waste and litter, was also positively tied to physical activity [63]. Thus, both maintenance of local spaces and the absence of litter contribute to the perception of appealing scenery. Although accessibility to various social resources is important, good maintenance of them, in terms of aesthetics, is even more important. That is, even if physical resources are close, if they are not aesthetically pleasing, children and families will not be motivated to use them. Importantly, neighborhood aesthetics are likely to provide children not only with access to play and physical activity, but also offer attractiveness and intellectual stimulation.

Stress is a third possible mechanism. Neighborhood disorder can contribute to emotional distress for children and their families, and the impact of neighborhood disorder on developmental outcomes and health is mediated by psychological and physiological distress [64,65,66,67]. Ultimately, chronic stressors in the environment may cause a psychological and physiological stress reaction which can negatively affect health and well-being. Children and families living in a disordered neighborhood may be exposed to chronic stressors including crime, neighborhood trouble, and harassment. Moreover, exposure to a disordered neighborhood (e.g., lower safety and social cohesion) may indirectly affect children’s mental health. This exposure may be mediated by maladaptive parental and family processes, thereby causing direct and adverse effects on children’s mental health induced by chronic stress. For instance, regarding neighborhoods with lower safety, physical and social aspects of the neighborhood, such as neighborhood stressors (e.g., crime, incivilities, trouble, harassment, and other potentially distressing signs of disorder), may influence child development. Certainly, family stress is a central predictor of behavioral problems in children [68,69]. Neighborhood disorder constitutes a risk factor that may increase stress for the child while potentially influencing parenting stress [70]. Through parenting stress, neighborhood disorder is often tied to negative parenting outcomes, including ineffective parenting practices, involvement, and monitoring [71]. Thus, living in a neighborhood with high levels of disorder could affect the ability to effectively parent, which, in turn, may increase parenting stress and result in more frustrated or angry reactive parenting behaviors. Despite the narrow range of neighborhood scores, in this study, there might be a greater correlation if a broader range of neighborhood types were examined. Additionally, neighborhood disorder has an indirect effect on children’s behavioral problems and cognitive development, transmitted through parenting quality and parenting stress [72]. In neighborhoods with lower social cohesion, instead of relying on neighborhood resources to offer support with child-rearing, parents exhibit a tendency to disengage from their neighborhoods to cope with its dangers. Additionally, a central mechanism of neighborhood disorder on children’s development is the cumulative effect of exposure to chronic stress. Indeed, children from disordered neighborhoods are more likely to be exposed to physical and psychosocial stress due to domestic disturbances. As chronic exposure to stressors accumulates, the physiological response systems built to handle sporadic but acute demands are overwhelmed, resulting in the disruption of the self-regulatory processes that allow children to cope with external demands [73,74]. Therefore, neighborhood disorder is likely to negatively influence child development.

The last possible mechanism is neighborhood collective efficacy, represented by social cohesion. Social cohesion is characterized by mutual trust and social support among community members [25]. In the present study, greater social cohesion was negatively associated with behavioral problems in children and positively associated with an increased likelihood of adequate social competence. This result is consistent with prior studies. For example, neighborhood social cohesion and collective efficacy have been recognized as factors that determine behavioral problems in children [75,76,77]. In addition, prior studies targeting adolescents have found neighborhood cohesion to be connected to various outcomes, including less antisocial behavior, improved physical health, and higher academic achievement [26,78,79]. Thus, neighborhood social cohesion is likely to protect against neighborhood-level risk factors. Indeed, neighborhood cohesion is linked to fewer adjustment problems by moderating neighborhood-level risk. For instance, the presence of cohesion among neighbors reduced behavioral problems in socioeconomically disadvantaged children [27]. Thus, social cohesion can mitigate harmful effects on health by moderating the influence of disadvantaged structural conditions [3,25]. In addition, residing in a neighborhood with a strong sense of social cohesion mitigated levels of adjustment problems among maltreated children [80]. Although children who are exposed to secure conditions develop the prosocial skills necessary to avert conflict and enable fulfilling relationships with others, children who are exposed to insecure conditions fail to establish supportive connections with those around them, thus reinforcing initial feelings of insecurity and unworthiness. Importantly, children who are exposed to uncertain conditions do not view parents as a source of safety and comfort; instead, maltreated children may consider their parental figures unable to protect them from harm [81]. To feel secure, sources of social support in extra familial contexts may be considered a central resource for such families. In contrast, children in high cohesion neighborhoods are more prone to view neighborhood adults as trusted figures that are able to protect them from danger [82]. Thus, neighborhood cohesion likely plays a protective role in reducing adjustment problems through alleviating neighborhood-level risk.

In the present study, even after adjusting for the impact of family socioeconomic factors, specific neighborhood features were likely to affect the behavioral outcomes of children in middle childhood. This finding indicates that features of one’s neighborhood environment are associated with improved mental health, independent of family socioeconomic factors. As mentioned previously, several mechanisms, including play and physical activities, intellectual stimulation, stress, and neighborhood collective efficacy may explain the association between environment characteristics and behavioral features, and may serve as key resources that protect children from disadvantaged families.

### Limitations

This study has several limitations. First, our results are not applicable to all families due to the risk of attrition bias. As mentioned in the Materials and Methods section, the response rate was 52.2%, and returning participants generally had a higher SES than non-returning participants, indicating a risk of attrition bias (i.e., social biases in responses). Thus, there is a possibility that our results could not accurately evaluate the precise mechanism of children with lower SES, and, as such, our analyses may underestimate the impact of SES. In addition, the study was conducted in a limited geographical area in a Japanese urban center. Future research would benefit from samples with higher retention rates (specifically lower SES participants), and greater demographic and clinical diversity.

Second, parents were the main data source in this study, which presents a risk of reporting bias. This potential bias can cause serious problems in interpreting the results [83,84,85]. Singular views toward neighborhood factors, child mental health functioning, and family SES factors (e.g., family income) may be skewed, positively or negatively, which could lead to deceptive findings. In this study, neighborhood characteristics were measured via parents’ own observations, which may not accurately mirror their true neighborhood conditions [3]. Unfortunately, we were unable to combine these perceived conditions with actual resources and problems; thus, future research should explore effective ways of assessing neighborhood settings, such as through geographic information [86]. In addition, future studies should consider incorporating both subjective and objective characteristics of the neighborhood environment to develop more valid and reliable measurements.

In addition, regarding child mental health functioning, we used parents as our key informants. SDQ scores were assessed by observation from children’s daily behavior. There are possible pitfalls in the interpretation of SDQ. One potential pitfall of SDQ is that it may underestimate child behavioral features if unobserved differences across children, such as underlying health or behavioral problems, are behind the variation. Incorporating observations from various caregivers, such as teachers, would offer a more balanced assessment of a child’s behavioral outcomes. For example, children’s parents and teachers may have different views on their children’s adaptive behavior. Many findings indicate several discrepancies among informants, including parents and teachers of children. These discrepancies are particularly noticeable between a child’s parents and teachers in terms of assessing the child’s psychological well-being [87]. The differences may reflect the child’s symptoms or the opportunity to observe them. For example, it is difficult for parents to distinguish between behaviors that reflect the underlying psychopathology and behaviors that reflect the immaturity of self-regulatory competence. Conversely, teachers have the advantage of being able to observe the behavior of many children all at once. In addition, behavioral problems may be more apparent at school than at home. Therefore, in future studies, reports from several different informants, including those from teachers and others, will be needed to more accurately assess how neighborhood factors affect children’s mental health. Accessing teacher reports could be especially important to assess and predict school maladjustment and mental health problems [88]. Additionally, several studies suggest that the combination of teacher and parent reports with independent assessments is more sensitive, offering a less biased result, than either assessment alone [89].

Moreover, regarding SES, arguments for the analysis of the complex associations between components of neighborhood factors, family SES factors, and child mental functioning would not be fully realized with data provided exclusively from parents. Additionally, the variables may influence each other. We excluded many family factors, such as cohesion, values, parenting practices, and functioning, and it is possible that parental education levels or other demographic information could have influenced their views of family aspects and children’s adaptive functioning. There are several mechanisms through which SES can influence the occurrence of behavioral problems. For instance, SES is related to the unequal distribution of material resources that might be used to support healthy child development, and which can also lead to material deprivation [90]. Ultimately, lower SES is likely to negatively affect developmental outcomes since parents living under such conditions are unable to provide the material resources necessary for optimal child development. It has been argued that social inequalities influence child development through not only the direct material path, but also through the indirect psychosocial path, depending on relative socioeconomic position [91,92]. Lower socioeconomic position can negatively influence developmental outcomes in children through the parents’ experiences of psychological distress; lower SES is negatively associated with parental mental health, which, as a result, may negatively affect parental functioning and parent–child interactions, in turn predicting mental health problems in children [93]. Of course, SES is not the only factor that determines the area in which a family lives, and we did not include the family’s reasons for residing in that particular neighborhood (e.g., availability of public transport, good access to parents’ workplaces, good quality of schools, and near other family members). In addition, prior studies suggested the importance of SES stability (e.g., income in this case); income averaged over the past five years would be a more useful measure in this regard [94]. Therefore, in future studies, more reliable SES indicators and additional potential family factors will be needed to more accurately assess in what way neighborhood factors influence children’s mental health functioning.

Third, we employed a cross-sectional study design. Although we controlled for the effect of family SES, there was some confusion surrounding the direction of causation due to selection bias. This happens to be one of the principal methodological limitations of interpreting statistical relations between neighborhood factors and health [82]. Thus, future research should consider observing trends or trajectories of child outcomes over a period of time, employing longitudinal designs.

Fourth, it is possible that we underestimated the true connection between neighborhood characteristics and child outcomes. Despite the narrow range of variables in this study (as shown by the narrow ranges of the neighborhood characteristics in Table 1), the differences were still apparent, and might even be more dramatic if a broader range of neighborhood types were examined. In addition, the association between dependent and independent variables can only be accurately evaluated using standard multiple regression if the relationships are linear in nature. In this study, we tested linear relationships for factors associated with child outcomes. Since there are many instances in the social sciences where non-linear relationships appear, the regression analysis might therefore underestimate the real relationship in question [95].

Lastly, we precluded school context variables from our study, which could have influenced child outcomes. Although school context variables could offer valuable clues into child outcomes, evidence implies that neighborhoods may be more reliable outcome predictors than school contexts [96]. Nevertheless, to develop more valid and reliable measurements, future research should include school context variables.

## 5. Conclusions

After adjusting for family socioeconomic variables, neighborhood characteristics were significantly associated with child behavior. That is, greater aesthetic quality, walking environment, availability of healthy foods, safety, and social cohesion were negatively associated with behavioral problems in children, and positively associated with social competence. Despite our study limitations, our results have significant implications. For instance, our findings provide evidence that neighborhood features influence middle childhood behaviors. Furthermore, comprehending the mechanisms present in these connections could contribute to developing effective interventions aimed at promoting child behavioral outcomes. Given that one’s neighborhood residence may be a possible socioeconomic health determinant, policy and programs that improve the physical environment in economically disadvantaged neighborhoods should be promoted. Ultimately, identifying where best to intervene and invest resources is vital; neighborhoods offer a potentially powerful platform to positively impact and advocate for child behavioral outcomes.

## Figures and Tables

**Table 1:** Descriptive statistics of all study variables.

	Range	Mean	SD	Alpha
Neighborhood environments				
Aesthetic quality	1–5	3.62	0.57	0.69
Walking environment	1–5	3.36	0.68	0.86
Availability of healthy foods	1–5	3.47	0.86	0.90
Safety	1–5	3.32	0.75	0.78
Violence	1–4	1.29	0.44	0.75
Social cohesion	1–5	3.43	0.66	0.89
Activities with neighbors	1–4	2.06	0.74	0.87
Child behaviors				
Externalizing problem behaviors	0–20	4.73	3.17	0.76
Internalizing problem behaviors	0–20	3.33	2.89	0.70
Prosocial behaviors	0–10	6.70	2.12	0.72

Note: neighborhood environments were assessed using the Neighborhood Scale and child behaviors were assessed using the Strengths and Difficulties Questionnaire. Abbreviations: Standard deviation (SD), Alpha (Cronbach’s alpha).

**Table 2:** Socioeconomic status of participants (*n* = 695).

	*n*	%
Annual household income (JPY)		
<6,000,000	354	50.9
6–9,000,000	190	27.3
≥9,000,000	135	19.4
Mother’s education level		
Compulsory education/upper secondary school	148	21.3
Up to four years at college/university	284	40.9
More than four years at college/university	256	36.8
Father’s education level		
Compulsory education/upper secondary school	184	26.5
Up to four years at college/university	104	15.0
More than four years at college/university	381	54.8

**Table 3:** Association between neighborhood characteristics and externalizing problems.

	Model 1							Model 2						
	B	95% CI		SE	β	*p*	R^2^	B	95% CI		SE	β	*p*	R^2^
		Min	Max						Min	Max				
Aesthetic quality	−0.162	−0.246	−0.079	0.043	−0.147	<0.001	0.022	−0.154	−0.240	−0.068	0.044	−0.140	<0.001	0.084
Walking environment	−0.070	−0.120	−0.019	0.026	−0.104	0.007	0.011	−0.071	−0.123	−0.020	0.026	−0.108	0.006	0.081
Availability of healthy foods	−0.087	−0.179	0.005	0.047	−0.071	0.065	0.005	−0.048	−0.143	0.047	0.048	−0.038	0.323	0.071
Safety	−0.072	−0.177	0.034	0.054	−0.051	0.183	0.003	−0.065	−0.174	0.044	0.056	−0.045	0.245	0.074
Violence	−0.107	−0.243	0.030	0.069	−0.059	0.124	0.003	−0.064	−0.201	0.073	0.070	−0.035	0.357	0.072
Social cohesion	−0.125	−0.214	−0.036	0.046	−0.105	0.006	0.011	−0.157	−0.249	−0.065	0.047	−0.128	0.001	0.086
Activities with neighbors	0.016	−0.050	0.081	0.033	0.018	0.640	0.001	−0.008	−0.074	0.058	0.034	−0.009	0.821	0.067

Note: Model 1 is unadjusted. Model 2 is adjusted for family socioeconomic status and child gender. Abbreviations: Unstandardized coefficient (B), confidence interval (CI), standard error (SE), standardized coefficient (β), *p*-value (*p*).

**Table 4:** Association between neighborhood characteristics and internalizing problems.

	Model 1							Model 2						
	B	95% CI		SE	β	*p*	R^2^	B	95% CI		SE	β	*p*	R^2^
		Min	Max						Min	Max				
Aesthetic quality	−0.184	−0.261	−0.107	0.039	−0.179	<0.001	0.032	−0.169	−0.253	−0.086	0.043	−0.162	<0.001	0.036
Walking environment	−0.081	−0.127	−0.035	0.023	−0.133	0.001	0.018	−0.076	−0.124	−0.027	0.025	−0.124	0.002	0.027
Availability of healthy foods	−0.131	−0.215	−0.047	0.043	−0.116	0.002	0.013	−0.103	−0.194	−0.012	0.046	−0.089	0.026	0.020
Safety	−0.124	−0.220	−0.028	0.049	−0.097	0.011	0.009	−0.088	−0.193	0.016	0.053	−0.067	0.097	0.017
Violence	0.067	−0.056	0.191	0.063	0.041	0.284	0.002	0.063	−0.066	0.193	0.066	0.038	0.338	0.014
Social cohesion	−0.118	−0.200	−0.036	0.042	−0.108	0.005	0.012	−0.116	−0.204	−0.028	0.045	−0.101	0.010	0.023
Activities with neighbors	−0.054	−0.114	0.005	0.030	−0.069	0.075	0.005	−0.054	−0.117	0.009	0.032	−0.068	0.091	0.018

Note: Model 1 is unadjusted. Model 2 is adjusted for family socioeconomic status and child gender. Abbreviations: Unstandardized coefficient (B), confidence interval (CI), standard error (SE), standardized coefficient (β), *p*-value (*p*).

**Table 5:** Association between neighborhood characteristics and child prosocial behaviors.

	Model 1							Model 2						
	B	95% CI		SE	β	*p*	R^2^	B	95% CI		SE	β	*p*	R^2^
		Min	Max						Min	Max				
Aesthetic quality	0.083	0.026	0.139	0.029	0.111	0.004	0.012	0.094	0.033	0.155	0.031	0.124	0.003	0.037
Walking environment	0.032	−0.002	0.066	0.017	0.072	0.063	0.005	0.043	0.007	0.079	0.018	0.096	0.018	0.034
Availability of healthy foods	0.024	−0.038	0.085	0.031	0.029	0.453	0.001	0.011	−0.055	0.078	0.034	0.013	0.738	0.025
Safety	0.058	−0.012	0.128	0.036	0.062	0.105	0.004	0.089	0.013	0.165	0.039	0.091	0.022	0.033
Violence	0.004	−0.087	0.094	0.046	0.003	0.935	0.001	−0.009	−0.104	0.086	0.048	−0.008	0.849	0.023
Social cohesion	0.068	0.008	0.127	0.030	0.085	0.027	0.007	0.087	0.023	0.151	0.033	0.104	0.008	0.035
Activities with neighbors	0.003	−0.041	0.047	0.022	0.005	0.894	0.001	0.015	−0.032	0.061	0.024	0.025	0.536	0.026

Note: Model 1 is unadjusted. Model 2 is adjusted for family socioeconomic status and child gender. Abbreviations: Unstandardized coefficient (B), confidence interval (CI), standard error (SE), standardized coefficient (β), *p*-value (*p*).

## Supplementary Materials

The following are available online at https://www.mdpi.com/1660-4601/17/15/5491/s1, Table S1: The items of the Strengths and Difficulties Questionnaire; Table S2: The items of the Neighborhood Scale; Table S3: Family socioeconomic status and neighborhood characteristics; Table S4: Family socioeconomic status, child gender, and child behaviors; Table S5: Correlations between family socioeconomic status and neighborhood characteristic variables.
