# Supplementary material for: The Relationship between Neighborhood Environment and Child Mental Health in Japanese Elementary School Students
Source: Int J Environ Res Public Health. 2020 Jul 29;17(15):5491. doi: 10.3390/ijerph17155491 (PMC7432741; doi:10.3390/ijerph17155491)
Supplement: Supplementary file 1 [file ijerph-17-05491-s001.pdf]

Table S1. The items of the Strengths and Difficulties Questionnaire.

Items	Not true	Somewhat true	Certainly true
Emotional symptoms			
3. Often complains of headaches, stomach-aches or sickness	0	1	2
8. Many worries or often seems worried	0	1	2
13. Often unhappy, depressed or tearful	0	1	2
16. Nervous or clingy in new situations, easily loses confidence	0	1	2
24. Many fears, easily scared	0	1	2
Conduct problems			
5. Often loses temper	0	1	2
7. Generally well behaved, usually does what adults request	2	1	0
12. Often fights with other children or bullies them	0	1	2
18. Often lies or cheats	0	1	2
22. Steals from home, school or elsewhere	0	1	2
Hyperactivity			
2. Restless, overactive, cannot stay still for long	0	1	2
10. Constantly fidgeting or squirming	0	1	2
15. Easily distracted, concentration wanders	0	1	2
21. Thinks things out before acting	2	1	0
25. Good attention span, sees work through to the end	2	1	0
Peer problems			
6. Rather solitary, prefers to play alone	0	1	2
11. Has at least one good friend	2	1	0
14. Generally liked by other children	2	1	0
19. Picked on or bullied by other children	0	1	2
23. Gets along better with adults than with other children	0	1	2

Prosocial behaviors			
1. Considerate of other people's feelings	0	1	2
4. Shares readily with other children, for example toys, treats, pencils	0	1	2
9. Helpful if someone is hurt, upset or feeling ill	0	1	2
17. Kind to younger children	0	1	2
20. Often offers to help others (parents, teachers, other children)	0	1	2

Table S2. The items of the Neighborhood Scale.

Items	Strongly disagree	Disagree	Neutral	Agree	Strongly agree
Aesthetic quality					
1. There is a lot of trash and litter on the street in my neighborhood.	5	4	3	2	1
2. There is a lot of noise in my neighborhood.	5	4	3	2	1
3. In my neighborhood the buildings and homes are well-maintained.	1	2	3	4	5
4. The buildings and houses in my neighborhood are interesting.	1	2	3	4	5
5. My neighborhood is attractive.	1	2	3	4	5
Walking environment					
1. My neighborhood offers many opportunities to be physically active.	1	2	3	4	5
2. Local sports clubs and other facilities in my neighborhood offer many opportunities to get exercise.	1	2	3	4	5
3. It is pleasant to walk in my neighborhood.	1	2	3	4	5
4. The trees in my neighborhood provide enough shade.	1	2	3	4	5
5. In my neighborhood it is easy to walk places.	1	2	3	4	5
6. I often see other people walking in my neighborhood.	1	2	3	4	5
7. I often see other people exercising (for example, jogging, bicycling, playing sports) in my neighborhood.	1	2	3	4	5
Availability of healthy foods					
1. A large selection of fresh fruits and vegetables is available in my neighborhood.	1	2	3	4	5
2. The fresh fruits and vegetables in my neighborhood are of high quality.	1	2	3	4	5
3. A large selection of low-fat products is available in my neighborhood.	1	2	3	4	5
Perception of safety					
1. I feel safe walking in my neighborhood, day or night.	1	2	3	4	5
2. Violence is not a problem in my neighborhood.	1	2	3	4	5
3. My neighborhood is safe from crime.	1	2	3	4	5
Social cohesion					
1. People around here are willing to help their neighbors.	1	2	3	4	5

2. People in my neighborhood generally get along with each other.	1	2	3	4	5
3. People in my neighborhood can be trusted.	1	2	3	4	5
4. People in my neighborhood share the same values.	1	2	3	4	5
Items	Never	Rarely	Sometimes	Often	–
Violence					
1. During the past 6 months, how often was there a fight in your neighborhood in which a weapon was used?	1	2	3	4	–
2. During the past 6 months, how often were there gang fights in your neighborhood?	1	2	3	4	–
3. During the past 6 months, how often was there a sexual assault or rape in your neighborhood?	1	2	3	4	–
4. During the past 6 months, how often was there a robbery or mugging in your neighborhood?	1	2	3	4	–
Activities with neighbors					
1. About how often do you and people in your neighborhood do favors for each other? By favors, we mean such things as watching each other's children, helping with shopping, lending garden or house tools, and other small acts of kindness.	1	2	3	4	–
2. When a neighbor is not at home or on vacation, how often do you and other neighbors watch over their property?	1	2	3	4	–
3. How often do you and other people in the neighborhood ask each other for advice about personal things such as child-rearing or job openings?	1	2	3	4	–
4. How often do you and people in your neighborhood have parties or other get-togethers where other people in the neighborhood are invited?	1	2	3	4	–
5. How often do you and other people in your neighborhood visit in each other's homes or speak with each other on the street?	1	2	3	4	–

Table S3. Family socioeconomic status and neighborhood characteristics.

	Aesthetic quality			Walking environment			Availability of healthy foods			Safety			Violence			Social cohesion			Activities with neighbors		
	Mean	*SD*	*p*	*Mean*	*SD*	*p*	*Mean*	*SD*	*p*	*Mean*	*SD*	*p*	*Mean*	*SD*	*p*	*Mean*	*SD*	*p*	*Mean*	*SD*	*p*
Annual household income (JPY)																					
< 6,000,000	3.54	0.55		3.31	0.67		3.37	0.85		3.20	0.77		1.31	0.44		3.38	0.68		2.02	0.74	
6 – 9,000,000	3.66	0.59	<0.001	3.41	0.69	0.087	3.54	0.86	0.008	3.41	0.74	<0.001	1.28	0.41	0.609	3.50	0.65	0.112	2.13	0.76	0.254
≥ 9,000,000	3.79	0.55		3.43	0.67		3.61	0.83		3.48	0.69		1.27	0.49		3.43	0.61		2.07	0.70	
Mother's education level																					
Compulsory education / upper secondary school	3.43	0.55		3.19	0.70		3.25	0.96		3.15	0.76		1.29	0.42		3.33	0.71		2.04	0.74	
Up to four years at college / university	3.60	0.57	<0.001	3.31	0.69	<0.001	3.45	0.83	<0.001	3.29	0.77	0.001	1.27	0.43	0.450	3.43	0.68	0.044	2.09	0.80	0.816
More than four years at college / university	3.76	0.55		3.51	0.62		3.60	0.80		3.45	0.71		1.32	0.45		3.50	0.60		2.07	0.66	
Father's education level																					
Compulsory education / upper secondary school	3.54	0.54		3.25	0.73		3.39	0.90		3.21	0.71		1.27	0.41		3.41	0.69		2.11	0.78	
Up to four years at college / university	3.49	0.59	<0.001	3.23	0.65	0.001	3.31	0.90	0.003	3.24	0.72	0.001	1.27	0.45	0.632	3.36	0.60	0.254	1.91	0.75	0.057
More than four years at college / university	3.71	0.56		3.45	0.64		3.58	0.78		3.43	0.74		1.30	0.44		3.47	0.64		2.09	0.70	

Note: Neighborhood characteristics were assessed by the Neighborhood Scale: Higher scores indicate greater aesthetic quality, walkability, availability of healthy foods, safety, violence, social cohesion, and activities with neighbors.

Table S4. Family socioeconomic status, child gender, and child behaviors.

	Externalizing problem behaviors			Internalizing problem behaviors			Prosocial behaviors		
	Mean	*SD*	*p*	*Mean*	*SD*	*p*	*Mean*	*SD*	*p*
Annual household income (JPY)									
< 6,000,000	5.05	3.26		3.54	3.07		6.70	2.13	
6 – 9,000,000	4.68	3.22	0.004	3.12	2.50	0.129	6.72	2.03	0.994
≥ 9,000,000	3.98	2.66		3.06	2.85		6.70	2.25	
Mother's education level									
Compulsory education / upper secondary school	5.31	3.37		3.55	3.10		6.80	2.08	
Up to four years at college / university	4.81	3.28	0.011	3.13	2.83	0.314	6.79	2.08	0.223
More than four years at college / university	4.34	2.86		3.40	2.81		6.50	2.17	
Father's education level									
Compulsory education / upper secondary school	5.35	3.34		3.36	2.96		6.68	2.07	
Up to four years at college / university	5.67	3.62	<0.001	3.67	2.86	0.376	6.53	2.36	0.746
More than four years at college / university	4.17	2.80		3.22	2.91		6.72	2.11	
Child's gender									
Male	5.32	3.21	<0.001	3.12	2.69	0.065	6.39	2.22	<0.001
Female	4.17	3.04		3.53	3.07		7.00	1.97	

Note: Child behaviors were assessed by the Strengths and Difficulties Questionnaire: Higher scores indicate higher levels of externalizing problems, internalizing problems, and prosocial behaviors.

Table S5. Correlations between family socioeconomic status and neighborhood characteristic variables.

	1		2		3		4		5		6		7		8		9		10	
	r	*p*	*r*	*p*	*r*	*p*	*r*	*P*	*r*	*p*	*r*	*p*	*r*	*p*	*r*	*p*	*r*	*p*	*r*	*p*
1. Annual household income	-	-																		
2. Mother's education level	0.255**	<0.001	-	-																
3. Father's education level	0.253**	<0.001	0.370**	<0.001	-	-														
4. Aesthetic quality	0.174	<0.001	0.213	<0.001	0.147	<0.001	-	-												
5. Walking environment	0.081	0.038	0.186	<0.001	0.136	<0.001	0.447	<0.001	-	-										
6. Availability of healthy foods	0.117	0.002	0.151	<0.001	0.110	0.004	0.361	<0.001	0.388	<0.001	-	-								
7. Safety	0.155	<0.001	0.148	<0.001	0.136	<0.001	0.441	<0.001	0.395	<0.001	0.336	<0.001	-	-						
8. Violence	-0.037	0.336	0.028	0.460	0.036	0.356	-0.206	<0.001	-0.091	0.018	-0.063	0.096	-0.384	<0.001	-	-				
9. Social cohesion	0.046	0.231	0.095	0.013	0.047	0.225	0.386	<0.001	0.384	<0.001	0.282	<0.001	0.434	<0.001	-0.105	0.006	-	-		
10. Activities with neighbors	0.040	0.308	0.011	0.772	0.001	0.970	0.164	<0.001	0.277	<0.001	0.154	<0.001	0.222	<0.001	-0.014	0.725	0.526	<0.001	-	-

Note: Neighborhood characteristics were assessed by the Neighborhood Scale: Higher scores indicate greater aesthetic quality, walkability, availability of healthy foods, safety, violence, social cohesion, and activities with neighbors.
